# Importance of lipid ratios for predicting intracranial atherosclerotic stenosis

**DOI:** 10.1186/s12944-020-01336-1

**Published:** 2020-07-04

**Authors:** Wen-Song Yang, Rui Li, Yi-Qing Shen, Xing-Chen Wang, Qing-Jun Liu, Hai-Yang Wang, Qi Li, Guo-En Yao, Peng Xie

**Affiliations:** 1 Department of Neurology, The First Affiliated Hospital of Chongqing Medical University, Chongqing, 400016 China; 2 NHC Key Laboratory of Diagnosis and Treatment on Brain Functional Diseases, The First Affiliated Hospital of Chongqing Medical University, Chongqing, 400016 China; 3 Division of Life Sciences and Medicine, Department of Neurology, The First Affiliated Hospital of USTC, University of Science and Technology of China, Hefei, Anhui 230001 P.R. China; 4 Department of Psychiatry, The First People’s Hospital of Yunnan Province, Kunming, 650032 Yunnan China; 5 Department of Neurology, Yongchuan Hospital of Chongqing Medical University, Chongqing, 402160 China; 6 Department of Neurology, First Affiliated Hospital, PLA General Hospital, Beijing, 100048 China

**Keywords:** Apo B/apo A-I, Intracranial atherosclerotic stenosis, Lipid ratios, Remnant cholesterol, Stroke, Atherosclerosis, Symptomatic

## Abstract

**Background:**

This study aims to investigate the association of lipid ratios with intracranial atherosclerotic stenosis (ICAS) in a Chinese population.

**Methods:**

This cross-sectional study included 658 consecutive patients with ischemic stroke. Intracranial and extracranial arteries were evaluated for atherosclerotic stenosis using digital subtraction angiography or computed tomography angiography. Lipid ratios [total cholesterol (TC)/high-density lipoprotein-cholesterol (HDL-C), triglycerides (TG)/HDL-C, low-density lipoprotein-cholesterol (LDL-C)/HDL-C, non-high-density lipoprotein-cholesterol (non-HDL-C)/HDL-C, remnant cholesterol (RC)/HDL-C, apolipoprotein B (apo B)/apolipoprotein A-I (apo A-I), and apo B/HDL-C] were calculated.

**Results:**

The TC/HDL-C, LDL-C/HDL-C, RC/HDL-C, non-HDL-C/HDL-C, apo B/HDL-C and apo B/apo A-I ratios (all *P* < 0.05) were significantly associated with ICAS but not with extracranial atherosclerotic stenosis after adjustment for confounding factors. Receiver operating characteristic (ROC) curves analysis revealed that the apo B/apo A-I ratio had the largest area under the ROC curve (AUC) among lipid levels alone and for lipid ratios (AUC = 0.588). Lipid ratios had higher AUC values than those for lipid levels alone for the identification of ICAS.

**Conclusion:**

The TC/HDL-C, LDL-C/HDL-C, RC/HDL-C, non-HDL-C/HDL-C apo B/HDL-C, and apo B/apo A-I ratios were significantly related to ICAS risk. Compared with the other variables tested, the apo B/apo A-I ratio appeared to be a better discriminator for identifying ICAS risk in stroke patients.

## Take home message

Lipid ratios could be more valuable than lipid levels alone for predicting ICAS risk, and sex-based differences were examined regarding the predictive accuracy of lipid profiles.

## Background

Intracranial atherosclerotic stenosis (ICAS) is a major cause of ischemic stroke worldwide [1, 2]. The prevalence of ICAS is relatively high in Asians, whereas extracranial atherosclerotic stenosis (ECAS) is more common in Caucasians. The exact causes for this distribution of cerebral atherosclerosis are not clear, but racial differences, socioeconomic status, and risk factors may contribute to this phenomenon [3]. Because of the detrimental effects of ICAS, a better understanding of its risk factors is particularly important.

The role of lipid parameters and lipid ratios in ICAS is controversial. Low-density lipoprotein cholesterol (LDL-C) is associated mainly with ECAS [4]. In Chinese populations, high-density lipoprotein-cholesterol (HDL-C) [5], non-high-density lipoprotein-cholesterol (non-HDL-C) [6] and total cholesterol (TC) [7] levels are associated with an increased risk of ICAS. One study based on a Korean population revealed the importance of hypercholesterolemia in ICAS in men [8]. Concerning lipid ratios, Park and colleagues indicated the importance of the apolipoprotein B/apolipoprotein A-I (apoB/apo A-I) ratio for ICAS [9]. Levels of HDL-C along with those of remnant cholesterol (RC) and a low apo B/apo A-I ratio are related to the prevention of the angiographic progression of ICAS [10]. Recently, a study found that acute ischemic stroke patients with ICAS have a higher ApoB/AI ratio was meaningfully higher than those without ICAS in both stroke group and non-stroke groups [11]. The LDL-C/HDL-C ratio is regarded as a marker for carotid intima-media thickness (CIMT) progression [12]. In addition, several lipid ratios (apo B/apo A-I, TC/HDL-C, LDL-C/HDL-C, and non-HDL-C/HDL-C) have shown clinically important correlations with coronary artery lesions, insulin resistance, and metabolic syndrome [13–15]. Moreover, higher LDL-C and lower HDL-C were associated with the presence of ICAS in a population-based study [16].

Lipid ratios are considered to be good parameters for identifying vascular diseases. Nevertheless, studies focusing on the association between lipid ratios and cerebral atherosclerosis are lacking. Additionally, the potential predictive importance of these lipid ratios in Chinese populations remains unclear. It is important to screen relevant applicable lipid ratios to identify high-risk populations. Therefore, this study compared the correlation between lipid ratios (TC/HDL-C, triglycerides (TG)/HDL-C, LDL-C/HDL-C, RC/HDL-C, non-HDL-C/HDL-C, apo B/HDL-C, and apo B/apo A-I) and the risk of cerebral atherosclerosis in patients with ischemic stroke.

## Methods

### Population and procedures

The study protocol was approved by the ethics committee of the First Affiliated Hospital of Chongqing Medical University (Chongqing, China). Written informed consent was obtained from all patients.

From November 2015 to January 2017, consecutive patients with transient ischemic attack or acute (< 7 days after onset) ischemic stroke admitted to the division within the First Affiliated Hospital of Chongqing Medical University were recruited prospectively into the study. All patients underwent cerebral digital subtraction angiography (DSA) or computed tomography angiography (CTA) to check for atherosclerotic lesions.

The exclusion criteria consisted of patients with contraindications to or who refused to undergo CTA or DSA; non-atherosclerotic arterial stenosis or totally occlusive stenosis in intracranial and extracranial vessels; and incomplete clinical information.

### Collection of clinical information

Information of each patient was acquired by completing a detailed questionnaire combined with a standardized interview. Information was obtained regarding demographics, hypertension, diabetes mellitus (DM), previous stroke, coronary heart disease, medical history, and current smoking. Blood pressure was determined with mercury manometers while the patient was seated after 10 min of rest. The parameter of “current smoking” was self-reported by patients who had smoked more than 100 cigarettes and smoked every day or some days now. The diagnoses of hypertension, DM, coronary heart disease, and previous stroke were determined according to those detailed in the *International Classification of Diseases* (9th revision).

### Laboratory measurements

Fasting blood samples were extracted and analyzed in the laboratory department of the First Affiliated Hospital. Lipid levels were measured using a fully automatic biochemistry analyzer (Cobas c701; Roche, Basel, Switzerland) and original reagents in the laboratory department. TC, TG, HDL-C, and LDL-C levels were measured using an enzymology assay. Apo A-I and apo B levels were measured by an immunoturbidimetric assay. The non-HDL-C level was defined as the TC level minus the HDL-C level [17]. The RC level was defined as the non-HDL-C level minus the LDL-C level [18]. Additionally, the TC/HDL-C, TG/HDL-C, LDL-C/HDL-C, RC/HDL-C, non-HDL-C/HDL-C, apo B/HDL-C, and apo B/apo A-I lipid ratios were calculated in this study.

### Assessment of cerebral atherosclerotic stenosis

ICAS was evaluated with the method used in the Warfarin–Aspirin Symptomatic Intracranial Disease Trial [19]. ECAS was evaluated based on the method used in the North American Symptomatic Carotid Endarterectomy Trial [20]. Intracranial vessels included the anterior, middle, and posterior cerebral arteries, the basilar artery, and intracranial portions of the vertebral and internal carotid artery. Extracranial vessels included extracranial segments of the vertebral artery and internal carotid artery. The presence of ICAS or ECAS was defined as stenosis ≥50% in large intracranial or extracranial vessels.

### Statistical analyses

Data were analyzed using SPSS 18.0 (Chicago, IL, USA) and MedCalc 11.4.2.0 (MedCalc, Ostend, Belgium). General characteristics were compared separately among patients between men and women, or among the groups of no cerebral atherosclerotic stenosis (NCAS), isolated ICAS, isolated ECAS, and ICAS combined ECAS. The frequency data were analyzed using the Student’s t-test or one-way analysis of variance (ANOVA) if the data obey the normal distribution and the variance is homogeneous. Mann–Whitney *U*-test was performed if the frequency data did not obey the normal distribution. The categorical variables were analyzed using the chi-square test. The results are expressed as the mean ± standard deviation for normal data or median and interquartile range for skewed data. Multiple logistic regression was carried out to evaluate the correlation of lipid ratios with ICAS and ECAS. Crude and adjusted odds ratios (ORs) with 95% confidence intervals (CIs) were obtained. Receiver operating characteristic (ROC) curves were created to assess whether variables could predict ICAS, and the results are expressed as the area under the curve (AUC). AUC values were compared using the Z-statistic. A value of *P* < 0.05 was considered significant.

## Results

Six hundred and fifty-eight patients (441 men and 217 women, mean age, 65.9 ± 11.2 years) with transient ischemic attack or acute ischemic stroke were included in the final analysis. Of the 658 patients analyzed, DSA was performed in 24 patients (3.6%) and the remaining 634 patients underwent CTA. The patients were divided into four subgroups as follows: No cerebral atherosclerotic stenosis (NCAS) (55.6%), Isolated ICAS (27.7%), Isolated ECAS (7.0%), and ICAS combined with ECAS (9.7%). The characteristics of the study participants in the groups of NCAS, isolated ICAS, isolated ECAS, and ICAS combined with ECAS groups were shown in Supplementary Table 1.

Sex-specific characteristics were shown in Table 1. Women were older and had lower diastolic blood pressure, and non-HDL-C/HDL-C, TC/HDL-C, RC/HDL-C, LDL-C/HDL-C, apo B/HDL-C, and apo B/apo A-I ratios than men. A significantly higher proportion of current smokers were men (*P* < 0.001), and their TC, HDL-C, and apo A-I levels were lower than those in women. Multivariable analysis of ICAS and (or) ECAS according to gender was shown in Supplementary Table 2.

**Table 1:** Characteristics of the study participants according to gender

Variables	Men (*n* = 441)	Women (*n* = 217)	*P* Value
Age, year	64.6 ± 11.2	68.5 ± 10.7	< 0.001
Systolic blood pressure, mmHg	152.8 ± 23.8	154.1 ± 26.0	0.522
Diastolic blood pressure, mmHg	88.7 ± 16.0	84.7 ± 14.6	0.002
Hypertension, n (%)	311 (70.5)	165 (76.0)	0.137
Diabetes mellitus, n (%)	144 (32.7)	74 (34.1)	0.711
Current smoking, n (%)	251 (56.9)	4 (1.8)	< 0.001
Previous stroke, n (%)	131 (29.7)	56 (25.8)	0.297
Coronary heart disease, n (%)	51 (11.6)	29 (13.4)	0.507
Number of ICAS lesions	0 (0–1)	0 (0–1)	0.771
NCAS	238 (54.0)	128 (59.0)	0.223
Isolated ICAS	115 (26.1)	67 (30.9)	0.196
Isolated ECAS	38 (8.6)	8 (3.7)	0.020
ICAS and ECAS	50 (11.3)	14 (6.5)	0.047
TC, mmol/L	4.35 ± 1.06	4.67 ± 1.10	< 0.001
TG, mmol/L	1.66 ± 1.23	1.62 ± 1.01	0.664
HDL-C, mmol/L	1.09 (0.94–1.24)	1.26 (1.09–1.51)	< 0.001
LDL-C, mmol/L	2.82 ± 0.91	2.97 ± 0.99	0.061
Non-HDL-C, mmol/L	3.16 (2.48–3.90)	3.21 (2.53–3.96)	0.384
RC, mmol/L	0.42 ± 0.40	0.37 ± 0.35	0.121
Apo A-I, g/L	1.22 ± 0.35	1.40 ± 0.27	< 0.001
Apo B, g/L	0.99 ± 0.53	0.96 ± 0.29	0.520
**Lipid Ratio**			
TC/HDL-C	4.12 ± 1.37	3.74 ± 1.22	0.001
TG/HDL-C	1.71 ± 1.77	1.45 ± 1.67	0.069
LDL-C/HDL-C	2.68 ± 1.06	2.39 ± 0.94	0.001
RC/HDL-C	0.44 ± 0.53	0.35 ± 0.55	0.036
Non- HDL-C/HDL-C	3.12 ± 1.37	2.73 ± 1.22	0.001
Apo B/HDL-C	2.25 (1.65–2.91)	1.87 (1.44–2.48)	< 0.001
Apo B/apo A-I	0.78 (0.60–0.98)	0.69 (0.53–0.86)	< 0.001

*ICAS* intracranial atherosclerotic stenosis, *ECAS* extracranial atherosclerotic stenosis, *NCAS* no cerebral atherosclerotic stenosis, *TC* total cholesterol, *TG* triglycerides, *LDL-C* low-density lipoprotein cholesterol, *HDL-C* high-density lipoprotein cholesterol, *RC* remnant cholesterol, *Non-HDL-C* non-high-density lipoprotein cholesterol, *Apo B* apolipoprotein B, *Apo A-I* apolipoprotein A-I

Results are expressed as mean ± standard deviation, median with interquartile range or n (%)

**Fig. 1:**
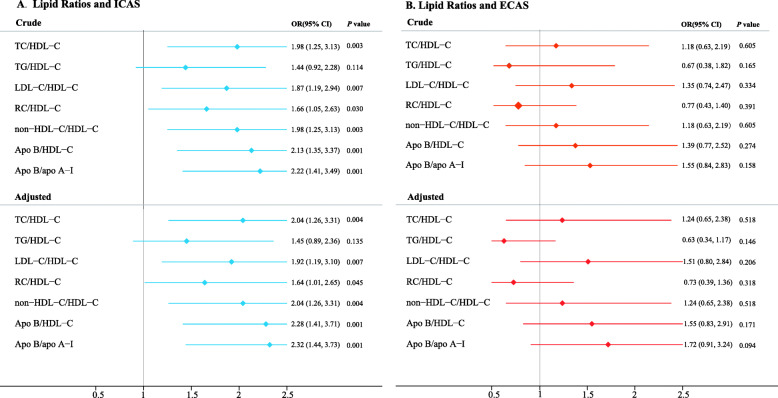
Logistic regression analysis of intracranial (**a**) and extracranial (**b**) atherosclerotic stenosis, among lipid ratios in the extreme quartiles of each evaluated variable. Data were adjusted for age, gender, current smoking, hypertension, diabetes mellitus, previous stroke, and coronary heart disease. *OR* odds ratio, *95%CI* 95% confidence interval, *TC* total cholesterol, *TG* triglycerides, *HDL-C* high-density lipoprotein cholesterol, *LDL-C* low-density lipoprotein cholesterol, *RC* remnant cholesterol, *Non-HDL-C* non-high-density lipoprotein cholesterol, *Apo B* apolipoprotein B, *Apo A-I* apolipoprotein A-I, *ICAS* intracranial atherosclerotic stenosis, *ECAS* extracranial atherosclerotic stenosis

Each lipid ratio was examined based on sex to assess its relationship with the risk of ICAS or ECAS. In this study, the lipid ratio in the fourth quartile was compared with that of the first quartile. The corresponding OR and 95% CI of lipid ratios (TC/HDL-C, LDL-C/HDL-C, TG/HDL-C, RC/HDL-C, non-HDL-C/HDL-C, apo B/apo A-I, and apo B/HDL-C) in the first quartile are listed (see Fig. 1). Compared with other lipid ratios, apo B/apo A-I showed the strongest relationship with ICAS after adjustment for potential confounding factors (first quartile vs. fourth quartile; [β] 0.84, [OR], 2.32; [95% CI], 1.44–3.73). Moreover, after adjustment for age, sex, current smoking, hypertension, DM, previous stroke, and coronary heart disease, this significant relationship was also observed for other lipid ratios (TC/HDL-C, [β] 0.71, [OR] 2.04, [95% CI] 1.26–3.31; RC/HDL-C, 0.49, 1.64, 1.01–2.65; non-HDL-C/HDL-C, 0.71, 2.04, 1.26–3.31; LDL-C/HDL-C, 0.65, 1.92, 1.19–3.10; apo B/HDL-C, 0.83, 2.28, 1.41–3.71; fourth quartile vs. first quartile; see Fig. 1a and Supplementary Table 3). These adjusted logistic regression analyses were repeated for ECAS, but no significant association was observed between lipid ratios and ECAS (see Fig. 1b).

**Fig. 2:**
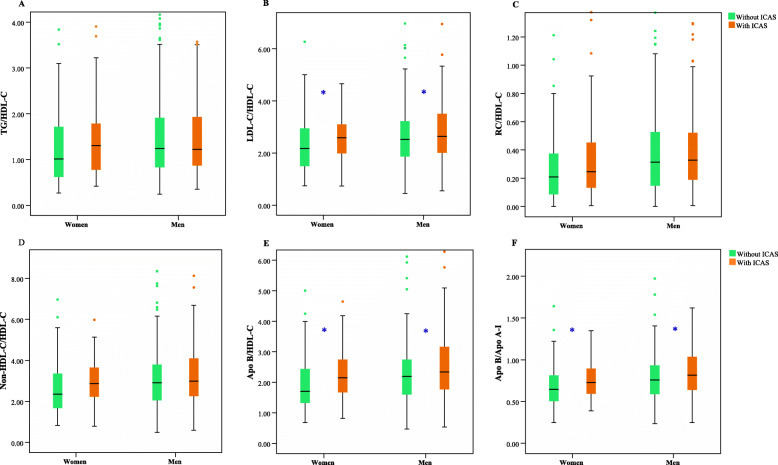
Box-plots of lipid ratios values and their association with ICAS in men and women. The total cholesterol (TC)/high-density lipoprotein cholesterol (HDL-C) (**a**), low-density lipoprotein cholesterol (LDL-C)/HDL-C (**b**), remnant cholesterol (RC)/HDL-C (**c**), non-high-density lipoprotein cholesterol (Non-HDL-C)/HDL-C (**d**), apolipoprotein B (Apo B)/HDL-C (**e**), and Apo B/apolipoprotein A-I (Apo A-I) (**f**) ratios are shown. *TG* triglycerides, *ICAS* intracranial atherosclerotic stenosis. *Significant difference at *P* < 0.05

Box-plots of the TC/HDL-C (see Fig. 2a), LDL-C/HDL-C (see Fig. 2b), RC/HDL-C (see Fig. 2c), Non-HDL-C/HDL-C (see Fig. 2d), Apo B/ HDL-C (see Fig. 2e), and Apo B/Apo A-I ratios (see Fig. 2f) were shown. Patients with ICAS had significantly higher LDL-C/HDL-C (see Fig. 2b), apo B/HDL-C (see Fig. 2e), and apo B/apo A-I ratios (see Fig. 2f) than those without ICAS in both sexes (*P* < 0.05). However, this difference was not observed for the TG/HDL-C (see Fig. 2a) and RC/HDL-C ratios (see Fig. 2c). A significant difference in the non-HDL-C/HDL-C ratio was observed only in men (see Fig. 2d). Because the result was similar to that of the non-HDL-C/HDL-C ratio, the TC/HDL-C ratio was not presented (see Fig. 2).

Analysis of ROC curves revealed that the apo B/apo A-I ratio exhibited the highest AUC value (0.588) for ICAS among all of the lipid levels alone and the lipid ratios (see Table 2). However, the apo B/apo A-I ratio exhibited the highest AUC value only in men but not in women (men: 0.579; women: 0.613). In women, the apo B/HDL-C ratio demonstrated an optimal AUC value (0.617) for predicting ICAS risk. Overall, lipid ratios had higher AUC values than those for the lipid levels alone for the identification of ICAS. Additionally, the AUC in women was higher than that in men for all lipid ratios analyzed.

**Table 2:** Comparison of AUC for each evaluated variable in predicting ICAS

	Total	Men	Women
	AUC(95% CI)	AUC(95%CI)	AUC(95%CI)
**Lipid measures**			
TC	0.543 (0.498–0.589)	0.537 (0.481–0.592)	0.562 (0.482–0.642)
TG	0.525 (0.480–0.570)	0.499 (0.444–0.554)	0.576 (0.500–0.652)
HDL-C	0.462 (0.417–0.507)	0.480 (0.426–0.535)	0.435 (0.356–0.514)
LDL-C	0.555 (0.510–0.601)	0.547 (0.491–0.603)	0.573 (0.493–0.653)
Non-HDL-C	0.559 (0.513–0.604)	0.543 (0.488–0.599)	0.590 (0.511–0.669)
RC	0.538 (0.493–0.583)	0.529 (0.475–0.584)	0.558 (0.481–0.635)
Apo A-I	0.456 (0.411–0.501)	0.465 (0.410–0.520)	0.424 (0.345–0.504)
Apo B	0.576 (0.531–0.622)	0.561 (0.505–0.617)	0.605 (0.527–0.683)
**Lipid ratios**			
TC/HDL-C	0.566 (0.521–0.611)	0.546 (0.491–0.601) *	0.608 (0.531–0.684)
TG/HDL-C	0.535 (0.490–0.579) *	0.511 (0.456–0.566) *	0.580 (0.503–0.657)
LDL-C/HDL-C	0.570 (0.525–0.615)	0.555 (0.499–0.611)	0.603 (0.526–0.680)
RC/HDL-C	0.544 (0.500–0.589)	0.530 (0.475–0.584)	0.575 (0.498–0.652)
Non- HDL-C/HDL-C	0.566 (0.521–0.611)	0.546 (0.491–0.601) *	0.608 (0.531–0.684)
Apo B/ HDL-C	0.578 (0.533–0.623)	0.562 (0.506–0.617)	0.617 (0.541–0.693)
Apo B/apo A-I	0.588 (0.543–0.633)	0.579 (0.523–0.634)	0.613 (0.536–0.690)

*TC* total cholesterol, *TG* triglycerides, *LDL-C* low-density lipoprotein cholesterol, *HDL-C* high-density lipoprotein cholesterol, *RC* remnant cholesterol, *Non-HDL-C* non-high-density lipoprotein cholesterol, *Apo B* apolipoprotein B, *Apo A-I* apolipoprotein A-I, *AUC* area under the curve; *95%CI* 95% confidence interval, *ICAS* intracranial atherosclerotic stenosis. * *P* < 0.05, AUC were compared with apo B/apo A-I ratio in lipid ratios

## Discussion

This study revealed that the TC/HDL-C, RC/HDL-C, LDL-C/HDL-C, non-HDL-C/HDL-C, apo B/HDL-C, and apo B/apo A-I ratios were significantly correlated with ICAS. Moreover, ROC analyses revealed that lipid ratios were better than lipid levels alone for predicting ICAS. The apo B/apo A-I ratio had a higher predictive value than that of other variables. This is the first study to present these relationships in a Chinese population.

The correlation between the apo B/apo A-I ratio and ICAS was established in a Korean population by Park and colleagues. They demonstrated that a higher apo B/apo A-I ratio could be a discriminator for ICAS rather than ECAS [9]. Sun et al. [11] found that apoB/AI ratio could be an independent factor for risk stratification of ICAS in both stroke patients and non-stroke controls, which is similar to the results of this study. Moreover, this ratio has also been shown to be superior to other ratios for the identification of coronary artery lesions in a Chinese population and coronary disease in a Swedish population [13, 21]. Thus, the apo B/apo A-I ratio is considered to be an excellent surrogate for the prediction of vascular disease risk. Nevertheless, few studies have focused on the apo B/HDL-C ratio. Maki and colleagues demonstrated that the apo B/apo A-I ratio can be a predictor of CIMT progression in vascular walls [22]. Biswas et al. [23] demonstrated that this ratio correlated well with the risk of coronary heart disease among Indian populations. Atherosclerosis is one of the main contributory indicator for coronary heart disease [24, 25]. Hence, the apo B/HDL-C ratio has been proposed to be a marker of atherosclerosis.

This study also identified the important predictive abilities of the apo B/HDL-C and apo B/apo A-I ratios for ICAS rather than ECAS. This phenomenon could be explained by the greater effect on antioxidant enzymes in ICAS than in ECAS [26]. In an experimental study, dyslipidemic mice were characterized by increased oxidation of apoB in the blood and impaired HDL-associated antioxidative defense [27]. Moreover, apoA-I is considered a marker of antioxidant and anti-inflammatory properties [28], and is closely associated with the patients with pre-existing ischemic stroke [29]. Thus, this deficit in antioxidant protection might be the pathomechanism of ischemic stroke in ICAS in patients with high apoB/apoA-I ratios [29].

As the crucial lipoprotein in intermediate-density lipoprotein (IDL), very low-density lipoprotein (VLDL), as well as LDL and apo B, can reflect the potential atherogenic lipoprotein particles in lipid metabolism [30]. Conversely, apo A-I is the main component in HDL-C, which has anti-atherogenic and anti-inflammatory potential [31]. HDL-C can reverse cholesterol transport. Apo A-I and HDL-C have valuable antioxidant capacities. Therefore, the apo B/HDL-C and apo B/apo A-I ratios can more comprehensively reflect atherogenicity and lower antioxidant capacity.

Non-HDL-C integrates multiple types of cholesterol, including IDL, LDL, VLDL, and lipoprotein (a) and can be determined simply by calculation (TC level minus the HDL-C level) [17]. Because of the protective role of HDL-C against cardiovascular diseases, non-HDL-C has an atherogenic effect in the circulation. This association between non-HDL-C and ICAS has been confirmed in a Chinese population [6]. However, the diagnostic ability of the non-HDL-C/HDL-C ratio for ICAS has not been investigated. Moreover, the non-HDL-C/HDL-C ratio possesses good predictive ability for some diseases. This ratio is more useful than the apo B/apo A-I ratio for identifying the metabolic syndrome in a Korean population. In addition, a large retrospective study demonstrated a positive correlation between insulin resistance and C-reactive protein levels [32]. Among individuals with obesity and insulin resistance syndromes, lower HDL-C and higher non-HDL-C levels demonstrated associations with coronary heart disease in regression models [33]. Moreover, non-HDL-C/HDL-C ratio is a strong indicator for predicting carotid atherosclerotic plaque in middle-aged postmenopausal women [34]. Besides, for the prediction of risk of cardiovascular diseases, this ratio is similar to the apo B/apo A-I ratio for DM patients [35]. Compared with traditional lipid variables, this ratio is more suitable for the estimation of arterial stiffness in a Chinese population [36]. In this study, the non-HDL-C/HDL-C ratio was associated with a two-fold risk of ICAS. Therefore, control of this ratio may be important for ICAS risk. The results of the TC/HDL-C ratio were similar to those of the non-HDL-C/HDL-C ratio.

RC comprises the TG-rich lipoproteins IDL, VLDL, and chylomicrons [18]. RC is regarded as a causal indicator of cardiovascular diseases [37, 38]. The diagnostic values of RC and the RC/HDL-C ratio have been identified simultaneously in Chinese patients with peri-procedural myocardial injury [39]. The RC/HDL-C ratio appears to be a useful tool for assessing the risk of ICAS. Compared with other important lipid ratios, only the RC/HDL-C ratio showed a slight association with ICAS in this study. The exact mechanism of action is not clear, but low-grade inflammation caused by RC could be one reason for this association. Subsequently, RC could enter vascular walls by infiltrating the endothelial barrier, and lead to the formation of foam cells by upregulating the expression of scavenger receptors [40].

### Study strengths and limitations

This study had several strengths. First, this study not only verified the data of other studies, but it also clarified the specific diagnostic value of lipid ratios upon ICAS. This study found that lipid ratios were better than routinely used lipid concentrations for predicting ICAS. Second, ROC curves indicated that the diagnostic ability of the apo B/apo A-I ratio surpassed those of all other ratios tested. This ratio was determined to be the best marker for ICAS risk. The lipid ratios indicated the balance between anti-atherogenic and pro-atherogenic mechanisms. The combined effects of lipid ratios could be more valuable than lipid levels alone. Third, this study suggested that lipid ratios had better predictive values than those of lipid levels alone for identifying ICAS risk. Finally, sex-based differences were examined regarding the predictive accuracy of lipid profiles. The apo B/apo A-I ratio had the highest AUC value for men, and the apo B/HDL-C ratio displayed the highest AUC value for women. One interpretation is that estrogen may exert an effect on lipid profiles, but the exact cause is not known, and further investigation is needed.

This study had five main limitations. First, data from a relatively small hospital-based population cannot be generalized to larger populations. Second, given the cross-sectional design and a relatively small cohort of patients with symptomatic ischemic stroke, a causal relationship between lipid ratios and ICAS could not be accurately ascertained. Third, this study focused on a Chinese population, therefore, the conclusions cannot be extrapolated to different ethnic groups. Fourth, cerebral atherosclerotic stenosis was evaluated using DSA and CTA. These methods have a high degree of accuracy for evaluating the severity of stenosis but are less accurate for cerebral atherosclerotic stenosis. Fifth, the absence of data such as insulin resistance, inflammatory indicators, menopausal status, the use of hypolipidemic drugs or anti-hypertensives drugs, apo CIII, apo-E, lipoprotein(a), and dietary habits may have a potential influence on outcomes.

## Conclusion

The TC/HDL-C, LDL-C/HDL-C, RC/HDL-C, non-HDL-C/HDL-C, and apo B/HDL-C ratios were significantly related to ICAS risk. Compared with the other variables tested, the apo B/apo A-I ratio appeared to be a better discriminator for identifying ICAS risk. Lipid ratios could be potential indicators for risk stratification of ICAS, and provide guidance in lipid-lowering therapy in patients with symptomatic ischemic stroke.

## Supplementary information

**Additional file 1.**

## Data Availability

The datasets used and/or analyzed during the current study are available from the corresponding author on reasonable request.

## Abbreviations

Apo A-I: Apolipoprotein A-I
Apo B: Apolipoprotein B
ICAS: Intracranial atherosclerotic stenosis
ECAS: Extracranial atherosclerotic stenosis
Non-HDL-C: Non-high-density lipoprotein cholesterol
RC: Remnant cholesterol
TC: Total cholesterol
TG: Triglycerides
LDL-C: Low-density lipoprotein cholesterol
HDL-C: High-density lipoprotein cholesterol
SE: Indicates standard error
*β*: Regression coefficients
OR: Odds ratio
95%CI: 95% confidence interval
AUC: Area under the curve
DSA: Digital subtraction angiography
CTA: Computed tomography angiography
CIMT: Carotid intima-media thickness
DM: Diabetes mellitus
IDL: Intermediate-density lipoprotein
VLDL: Very low-density lipoprotein
NCAS: No cerebral atherosclerotic stenosis
