# Supplementary material for: Importance of lipid ratios for predicting intracranial atherosclerotic stenosis
Source: Lipids Health Dis. 2020 Jul 4;19:160. doi: 10.1186/s12944-020-01336-1 (PMC7335436; doi:10.1186/s12944-020-01336-1)
Supplement: Supplementary file 1 — Additional file 1. [file 12944_2020_1336_MOESM1_ESM.docx]

**Supplementary Table 1** Characteristics of the study participants among the groups of NCAS, isolated ICAS, isolated ECAS, and ICAS combined ECAS.

| Variables | NCAS  (n=366, 55.6%) | Isolated ICAS (n=182, 27.7%) | Isolated ECAS (n=46, 7.0%) | ICAS combined ECAS (n=64, 9.7%) | *P* Value |
| --- | --- | --- | --- | --- | --- |
| Age, year | 65.4±11.4 | 65.6±11.0 | 66.4±10.8 | 69.1±10.7 | 0.098 |
| Gender, male | 238(65.0) | 115(63.2) | 38(82.6) | 50(78.1) | 0.015 |
| Systolic blood pressure, mmHg | 151.6±24.4 | 155.3±24.6 | 149.8±23.8 | 158.8±24.6 | 0.067 |
| Diastolic blood pressure, mmHg | 87.7±16.1 | 88.2±15.1 | 82.8±14.0 | 86.6±15.7 | 0.193 |
| Hypertension, n (%) | 249 (68.0) | 142 (78.0) | 30 (65.2) | 55 (85.9) | 0.004 |
| Diabetes mellitus, n (%) | 107 (29.2) | 65 (35.7) | 17 (37.0) | 29 (45.3) | 0.054 |
| Current smoking, n (%) | 137(37.4) | 65 (35.7) | 24 (52.2) | 29 (45.3) | 0.131 |
| Previous stroke, n (%) | 88 (24.0) | 61 (33.5) | 14 (30.4) | 24 (37.5) | 0.038 |
| Coronary heart disease, n (%) | 41 (11.2) | 22 (12.1) | 9 (19.6) | 8 (12.5) | 0.443 |
| TC, mmol/L | 4.39±1.08 | 4.50±1.10 | 4.45±0.89 | 4.66±1.08 | 0.272 |
| TG, mmol/L | 1.67±1.22 | 1.64±0.98 | 1.33±0.58 | 1.78±1.51 | 0.214 |
| HDL-C, mmol/L | 1.15 (0.96-1.36) | 1.14(0.95-1.28) | 1.16(0.99-1.37) | 1.10(0.98-1.26) | 0.326 |
| LDL-C, mmol/L | 2.79±0.92 | 2.94±0.98 | 2.90±0.86 | 3.09±0.95 | 0.007 |
| Non-HDL-C, mmol/L | 3.06 (2.44-3.90) | 3.31(2.53-3.94) | 3.36 (2.79-3.63) | 3.44(2.96-4.10) | 0.035 |
| RC, mmol/L | 0.40±0.39 | 0.41±0.32 | 0.31±0.18 | 0.45±0.51 | 0.010 |
| Apo A-I, g/L | 1.30±0.39 | 1.24±0.24 | 1.28±0.27 | 1.25±0.24 | 0.187 |
| Apo B, g/L | 0.94±0.43 | 1.00±0.43 | 0.94±0.24 | 1.16±0.76 | 0.006 |
| **Lipid Ratio** |  |  |  |  |  |
| TC/HDL-C | 3.89±1.31 | 4.09±1.26 | 3.78±1.12 | 4.43±1.66 | 0.012 |
| TG/HDL-C | 1.64±1.74 | 1.60±1.29 | 1.17±0.67 | 1.95±2.94 | 0.002 |
| LDL-C/HDL-C | 2.49±1.01 | 2.68±1.03 | 2.51±1.02 | 2.90±1.06 | 0.010 |
| RC/HDL-C | 0.41±0.51 | 0.41±0.42 | 0.27±0.19 | 0.52±0.99 | <0.001 |
| Non- HDL-C/HDL-C | 2.89±1.31 | 3.09±1.26 | 2.78±1.12 | 3.43±1.66 | 0.012 |
| Apo B/HDL-C | 2.03 (1.51-2.68) | 2.16 (1.68-3.01) | 2.19 (1.44-2.62) | 2.51 (1.81-3.15) | 0.002 |
| Apo B/apo A-I | 0.71 (0.56-0.88) | 0.77 (0.60-0.98) | 0.81 (0.55-0.96) | 0.86 (0.68-1.02) | <0.001 |

*ICAS* indicates intracranial atherosclerotic stenosis, *ECAS* extracranial atherosclerotic stenosis, *NCAS* no cerebral atherosclerotic stenosis, *TC* total cholesterol, *TG* triglycerides, *LDL-C* low-density lipoprotein cholesterol, *HDL-C* high-density lipoprotein cholesterol, *RC* remnant cholesterol, *Non-HDL-C* non-high-density lipoprotein cholesterol, *Apo B* apolipoprotein B, *Apo A-I* apolipoprotein A-I.

Results are expressed as mean±standard deviation, median with interquartile range or n (%).

**Supplementary Table 2** Multivariable analysis of ICAS and (or) ECAS according to gender

| Variables | β | SE | OR value | 95% CI | *P* Value |
| --- | --- | --- | --- | --- | --- |
| NCAS | Reference |  |  |  |  |
| Isolated ICAS | -0.03 | 0.23 | 0.97 | 0.62-1.52 | 0.892 |
| Isolated ECAS | 0.74 | 0.74 | 2.10 | 0.85-5.15 | 0.160 |
| ICAS and ECAS | 0.71 | 0.71 | 2.03 | 0.97-4.25 | 0.061 |

*β*indicates regression coefficients, *SE* standard error, *ICAS* intracranial atherosclerotic stenosis, *ECAS* extracranial atherosclerotic stenosis, *NCAS* no cerebral atherosclerotic stenosis.

Adjusted for age, current smoking, hypertension, diabetic mellitus, previous stroke, and coronary heart disease.

**Supplementary Table 3** Results of univariate and multivariate linear regression for ICAS and ECAS

| Variables | **Univariate Analysis** | | | **Multivariate Analysis**^#^ | | |
| --- | --- | --- | --- | --- | --- | --- |
|  | β | SE | P value | β | SE | P value |
| **ICAS** |  |  |  |  |  |  |
| TC/HDL-C | 0.68 | 0.23 | 0.003 | 0.71 | 0.25 | 0.004 |
| TG/HDL-C | 0.37 | 0.23 | 0.114 | 0.37 | 0.25 | 0.135 |
| LDL-C/HDL-C | 0.63 | 0.23 | 0.007 | 0.65 | 0.24 | 0.007 |
| RC/HDL-C | 0.51 | 0.24 | 0.030 | 0.49 | 0.25 | 0.045 |
| Non- HDL-C/HDL-C | 0.68 | 0.23 | 0.003 | 0.71 | 0.25 | 0.004 |
| Apo B/HDL-C | 0.76 | 0.23 | 0.001 | 0.83 | 0.25 | 0.001 |
| Apo B/apo A-I | 0.80 | 0.23 | 0.001 | 0.84 | 0.24 | 0.001 |
| **ECAS** |  |  |  |  |  |  |
| TC/HDL-C | 0.16 | 0.32 | 0.605 | 0.22 | 0.33 | 0.518 |
| TG/HDL-C | -0.41 | 0.29 | 0.165 | -0.46 | 0.32 | 0.146 |
| LDL-C/HDL-C | 0.30 | 0.31 | 0.334 | 0.41 | 0.32 | 0.206 |
| RC/HDL-C | -0.26 | 0.30 | 0.391 | -0.32 | 0.32 | 0.318 |
| Non- HDL-C/HDL-C | 0.16 | 0.32 | 0.605 | 0.22 | 0.33 | 0.518 |
| Apo B/HDL-C | 0.33 | 0.30 | 0.274 | 0.44 | 0.32 | 0.171 |
| Apo B/apo A-I | 0.44 | 0.31 | 0.158 | 0.54 | 0.32 | 0.094 |

*β*indicates regression coefficients, *SE* standard error, *TC* total cholesterol, *TG* triglycerides, *HDL-C* high-density lipoprotein cholesterol, *LDL-C* low-density lipoprotein cholesterol, *RC* remnant cholesterol, *Non-HDL-C* non-high-density lipoprotein cholesterol, *Apo B* apolipoprotein B, *Apo A-I* apolipoprotein A-I, *ICAS* intracranial atherosclerotic stenosis, *ECAS* extracranial atherosclerotic stenosis.

^#^Adjusted for age, gender, current smoking, hypertension, diabetes mellitus, previous stroke, and coronary heart disease.
